# Supplementary material for: Estimating effects of parents’ cognitive and non-cognitive skills on offspring education using polygenic scores
Source: Nat Commun. 2022 Aug 23;13:4801. doi: 10.1038/s41467-022-32003-x (PMC9399113; doi:10.1038/s41467-022-32003-x)
Supplement: Supplementary file 1 — Supplementary Information [file 41467_2022_32003_MOESM1_ESM.pdf]

Estimating effects of parents' cognitive and non-cognitive skills on offspring education using polygenic scores

Supplementary Information

Supplementary Notes 3

Supplementary Note 1: Deviation from pre-registered methods 3

Supplementary Note 2: Meta-analyses of Cognitive Performance and Educational Attainment GWAS 4

Supplementary Note 3: Comparison of demographic and early-life characteristics of the adopted and non-adopted samples 5

Supplementary Note 4: Methods for simulating genetic and phenotypic data in the presence of different biases and components 7

Supplementary Note 5: Comparison of two implementations of the sibling design using simulation 11

Supplementary Note 6: Comparison of sibling, adoption, and non-transmitted allele designs in presence of simulated components and biases 12

Supplementary Figures 15

Supplementary Figure 1. GWAS-by-subtraction Genomic-SEM model, reproduced from Demange et al. 2021 15

Supplementary Figure 2. Birthplace coordinates for adoptees and non-adopted individuals 16

Supplementary Figure 3. All direct and indirect genetic effect estimates 17

Supplementary Figure 4. Population genetic effects 18

Supplementary Figure 5. Ratios indirect effect/population effect 19

Supplementary Figure 6. Effect of the *NonCog* and *Cog* polygenic score on educational outcomes in monozygotic and dizygotic twins in TEDS and NTR 20

Supplementary Figure 7. Estimated effect of NonCog and Cog PGS on Educational Attainment in UK Biobank depending on the number of siblings (adopted or full-siblings) of the individual 21

Supplementary Figure 8. Estimation of the direct and parental indirect genetic effects in simulated data with different implementations of the sibling design 22

Supplementary Figure 9. Simulation results: Comparison of sibling, adoption, and non-transmitted allele designs in presence of components and biases 23

Supplementary References 24

Supplementary Notes

Supplementary Note 1: Deviation from pre-registered methods

To correct for family structure in our trio data in NTR, we planned to use the gee function in R. To correct for additional shared factors in the sibling design, we planned to use the lme function in R to specify a random intercept for family (as done by [1]). However, the gee function led to convergence issues when bootstrapping. Additionally, simulations showed that the use of a mixed model (lme or lmer commands in R) in the sibling design leads to underestimation of indirect genetic effects, and underestimation of the direct genetic effects in the non-transmitted alleles design (see Supplementary Note 5). Hence, we use linear models in the sibling and non-transmitted allele design (lm command in R), and we bootstrapped standard errors (see Methods in the main manuscript).

Supplementary Note 2: Meta-analyses of Cognitive Performance and Educational Attainment GWAS

Before performing GWAS-by-subtraction, we ran a GWAS of Educational Attainment and Cognitive Performance in UKBiobank (polygenic score sample left-out). Genetic associations were obtained using fastGWA and controlling for age (Data-Field 21022), sex, array and the 25 first principal component analyses, for 1,246,531 SNPs (Hapmap 3 SNPs).
We then meta-analysed our UKB EA GWAS (N=388,196) with the EA GWAS by Lee et al excluding 23andMe, UK Biobank and NTR cohorts (N=318,916) using the METAL software. We included SNPs with sample-size > 500,000 and MAF > 0.005. We did not apply genomic control, but following Lee et al. we inflated the standard errors from the meta-analysis by the square root of the LD score intercept (1.223, SE=0.0223). After inflation of the SEs, we found a SNP heritability of 0.1006 (SE=0.0027) with a LD score intercept of 0.9783 (SE=0.0187).
We meta-analysed our UKB CP GWAS (N=202,815) with CP GWAS by Trampush et al (N=35,298) using the METAL software. We included SNPs with sample-size > 100,000 and MAF > 0.005. We found a SNP heritability of 0.1858 (SE=0.0064) with LDSC. We did not apply genomic control, and because the LD score intercept was acceptable (1.055, SE=0.0118), we did not inflate standard errors.

Supplementary Note 3: Comparison of demographic and early-life characteristics of the adopted and non-adopted samples

In the adoption design, indirect genetic effects are inferred by subtracting polygenic score associations estimated in a sample of adoptees away from those estimated in a non-adopted control group. When taking the difference, it is important that the groups are similar in characteristics other than genetic relatedness to their parents. We explored this empirically by comparing demographic and early-life characteristics of adoptees and nonadopted individuals in the UK Biobank.

Supplementary Data 11A displays results from our comparison of *NonCog* PGS, *Cog* PGS, birthweight, and educational attainment. We observed significant but small differences between the groups in their mean *NonCog* and *Cog* PGS as well as educational attainment (Cohen's d < 0.15). We do not observe differences in the variances of these measures between the two groups.

We observed differences in birthweight, with adopted individuals being lighter at birth than the non-adopted individuals (mean = 3.12kg vs 3.33kg, Cohen's d=0.31). The variance in birthweight was also significantly different, with more variance in the adoptee group (0.57 vs 0.45). However, missingness of birthweight data is severe in both groups, but particularly adoptees (72% of missing data among adoptees, 43% in the non-adopted group).

We investigated whether the birthplaces of adoptees and non-adoptees were clustered differently. If so, this could mean that population stratification effects are not consistent across the groups. Hence, we performed k-means clustering on the UK Biobank's east/west birthplace coordinates, separately for adoptees and non-adopted individuals. Without a hypothesis for the number of geographical clusters, we used different numbers of clusters (k), and then plotted the within-cluster sum of squares according to k. For both adopted and non-adopted groups, the best number of clusters was 4, indicated by the location of the bend in the plot. As shown in **Supplementary Figure 2 (below)**, the pattern of clustering was also the same between the groups, with the clusters reflecting the regions of Wales, the South, the North, and the Midlands.

Next, we compared the proportions of adopted and non-adopted individuals born in each UK region (see **Supplementary Data 11B**). Adoptees were less likely to have been born in the Midlands and more likely to have been born in the South, but these differences were small. Since there could be differences between the groups in location of birth *within* each region, we also compared the cluster means for the two groups. There were only small differences. The largest discrepancy was that adoptees from the north were born further south than the non-adopted northerners (northern coordinate 1.93 vs 1.99).

Notably, the interpretability of these analyses are hindered by data limitations. A large fraction of birthweight data was missing (72% missingness for the adoptee group). Also, birthplace was self-reported, so could be inaccurate, particularly for adoptees.

Overall, the key variables under study are well-matched between the adopted and non-adopted groups. There may be stronger systematic differences relating to birthweight and geography (as well as unobserved variables). Differences between the adopted and non-adopted groups are more of a concern for estimating indirect genetic effects than direct genetic effects, since the former but not the latter is based on comparison between groups. Direct genetic effects estimated using this design can be interpreted specifically for the adoption sample, whereas indirect genetic effect estimates have a more ambiguous interpretation since they are based on two different groups.

Supplementary Note 4: Methods for simulating genetic and phenotypic data in the presence of different biases and components

We simulate data introducing various potential components and biases, and then fit all models used throughout the paper to identify how the estimated parental indirect effect changes in the presence of these factors.

We simulate **genotype data** for 20,000 families. Each family includes a mother, a father, a focal offspring, a child sibling, and an adopted child sibling. The adoptee genotypes are drawn from another simulated dataset of biological parents, independent of the focal families. Therefore, the total sample size including the main families plus biological parents of adoptees is (20,000 x 5) + 20,000 = 120,000 individuals. Genotypes are simulated as 100 bi-allelic SNP calls, using the 'coin flipping' function in R *rbinom()*. For individuals in the parent generation, probability values for SNPs are defined by minor allele frequencies (simulated as deviates of the uniform distribution between .1 and .5). For offspring of these individuals, probability values for SNPs are defined as each parental genotype divided by 2.

We then simulate **'true' SNP effects**, drawn from a normal distribution. We use these true SNP effects to simulate **'true' genetic scores** for the mothers, fathers, and biological and adopted offspring. The true SNP effects are the same for all individuals and for all sub-populations. True genetic scores are used to simulate phenotypes.

In addition to 'true' polygenic scores, we create more realistic **'GWAS-based' polygenic scores** for all individuals by weighting their genotypes by **GWAS SNP effects**. We define GWAS SNP effects as true SNP effects with added error, and calculate them as sqrt(.2)*true effects + sqrt(.8)*error, the error following a normal distribution (this differs when simulating population stratification, see below). GWAS effects are the same for all individuals and sub-populations. GWAS-based polygenic scores are used to estimate direct and indirect effects. We also tested how sensitive the estimates from the three designs are to the amount of noise introduced in the GWAS effects, and found that this only matters for assortative mating (see assortative mating results below on page 13).

We simulate nine offspring phenotypes influenced by different factors:

i) direct genetic effects only,
ii) direct and indirect parental genetic effects (maternal and paternal),
iii) indirect parental genetic effects plus a prenatal indirect maternal genetic effect,
iv) indirect sibling genetic effect,
v) indirect parental genetic effects and an indirect sibling genetic effect,
vi) assortative mating,
vii) assortative mating and indirect parental genetic effects,
viii) population stratification,
ix) population stratification and indirect parental genetic effects.

Having simulated the nine phenotypes as detailed further below, we use **three designs** (sibling, adoption, non-transmitted allele, explained in the main article) to estimate indirect parental genetic effects on each phenotype. This allows us to evaluate how designs are affected by the components (prenatal and postnatal parental indirect genetic effects) and biases (sibling indirect genetic effects, assortative mating, population stratification). We repeated the simulation 100 times.

Note that these simulations are to illustrate how designs are affected by the components and biases. Effect sizes for each bias/component are not intended to represent true effects and as such are somewhat arbitrary. Additionally, by necessity we make certain untested assumptions. For example, indirect genetic effects are assumed to be equal between all siblings (i.e., no birth order effects or different effects for adoptive siblings), and population stratification and assortative mating are assumed to operate equally among biological and adoptive parents.

For the simulation results, see the below text under the heading *'Comparison of sibling, adoption, and non-transmitted allele designs in presence of simulated components and biases'*, and Supplementary Figure 9. The complete simulation code is available on GitHub[2].

Simulation details for the nine phenotypes

i) <u>Direct genetic effects</u>

We simulate child phenotypes influenced by direct genetic effects only, such that

$$y = var(g).x + e \quad \textbf{(1)}$$

Where y is the child phenotype, x is the true genetic score of the child, var(g) is the variance explained by the true genetic score and e is the residual error (explaining the rest of the variance).

Parental phenotypes used below are also simulated this way (i.e., influenced by own genotype plus environment/error).

ii) <u>Indirect parental genetic effects</u>

We simulate child phenotypes influenced by direct genetic effects and indirect parental genetic effects such that

$$y = var(g).x + var(mother).y_{mother} + var(father).y_{father} + e \quad \textbf{(2)}$$

Where y is the child phenotype, x is the true genetic score of the child, var(g) is the variance explained by the true genetic effect, y_{mother} and y_{father} are the parental phenotypes, $var(mother)$ and $var(father)$ are the variance explained by parental phenotypes, and e is the residual error (explaining the rest of the variance).

iii) Prenatal and postnatal indirect parental effects

We simulate child phenotypes influenced by direct genetic effects and prenatal and postnatal indirect parental genetic effects such that

$$y_{adoptee} = var(g).x + var(prenatal).y_{biological\ mother} + var(postnatal).y_{adoptive\ mother} + var(father).y_{father} + e \quad \textbf{(3)}$$

$$y_{non-adopted} = var(g).x + var(prenatal).y_{mother} + var(postnatal).y_{mother} + var(father).y_{father} + e \quad \textbf{(4)}$$

iv) Indirect sibling genetic effects &

v) Indirect sibling and parental indirect genetic effects

After simulating all sibling phenotypes with only direct effects or with direct and indirect parental genetic effects, we simulate indirect genetic effects operating among three siblings in each family: individual 1, a biological sibling, and an unrelated adopted sibling. First, we create a matrix of sibling effects in which every effect is of the same magnitude (all siblings have an equal effect on each other regardless of adoption status, an implicit assumption), with zeros on the diagonal. To account for feedback effects (e.g., sibling 1 influences sibling 2, who influences sibling 1; this changes the coefficients of a variable on its own errors), we subtract the sibling effect matrix from an identity matrix and take its inverse. We then take the matrix product of the matrix with sibling effects and the simulated sibling data to introduce the simulated mutual sibling effects into the data.

vi) Assortative mating

vii) Assortative mating and parental indirect genetic effects

Genetic assortative mating occurs when individuals with similar phenotypes mate more frequently than would be expected under a random mating scenario, and these phenotypes are heritable. To simulate assortment, we re-create offspring genotypes and polygenic scores after matching parents together systematically (instead of randomly as above). We first create phenotypes for the parents (based on true genetic score plus noise), rank the mothers and fathers by phenotype, and match couples according to rank (i.e., mothers with higher phenotypic values match with fathers with higher phenotypic values). Since mating does not perfectly track with phenotypic rank, we add noise to the ranking of mothers and fathers prior to matching, following a chosen phenotypic correlation. Offspring genotypes are then simulated as random draws from the matched couples' genotypes. Assortment is simulated to be the same strength for adoptees' and nonadoptees' parents, and we simulate random placement by un-ranking adoptees before matching them to adoptive families.

viii) Population stratification

ix) Population stratification and parental indirect genetic effects

Population stratification can be conceptualized as systematic differences in allele frequencies between sub-populations. These frequency differences cause confounding in genetic studies when phenotypes also differ between sub-populations. We simulate such sub-populations in both the GWAS discovery and target PGS analyses samples. We first create new genotypes in

two groups, drawing upon two different sets of simulated minor allele frequency distributions. We also define a phenotypic difference between these two groups, by including an 'environmental confounding' parameter which is noise with a different mean phenotype for the two sub-populations. We then run a single GWAS in these two populations. We create phenotypes and polygenic scores (based on the GWAS results) in a target sample of families, comprising the same two sub-populations present in the GWAS. Our simulation allows for adoptees to be matched with adoptive parents both within- and between- sub-populations. We report results from a simulation with adoptees matched with adoptive parents within the same sub-population.

Supplementary Note 5: Comparison of two implementations of the sibling design using simulation

In the sibling design presented by Selzam et al. (2019), indirect genetic effects are estimated by subtracting the within-sibling estimate from the between-sibling estimate (indexed using the average polygenic score for each sibling pair). However, the between-sibling effect is not necessarily the appropriate quantity to use [3]. An alternative is to subtract the within-sibling estimate from an estimate of the population effect obtained in a separate regression analysis using population data and ignoring family clustering. This approach was used in a recent within-sibling GWA study [4].

To ensure that we contrast our direct genetic effects with the appropriate quantity for accurate estimation of indirect genetic effects, we use simulated data to assess the use of the between-sibling effect and the population effect. Results are presented below in **Supplementary Figure 8**. From these simulations, it appears that contrasting the direct effects with the between-sibling effects leads to an overestimation of indirect parental genetic effects. Contrasting direct effects with population effects results in accurate estimation of indirect genetic effects. Consequently, we use this approach in our main analyses and simulations. Therefore, our model differs slightly from the Selzam et al. analyses.

Also notable is that, whilst the Selzam et al. article (and [5]) uses a different term – *passive gene-environment correlation* – the effect being estimated is a parental indirect genetic effect. Passive gene-environment correlation refers to how the genes that parents pass on to their children may also influence how they provide the rearing environment (Plomin et al. 1977).

Supplementary Note 6: Comparison of sibling, adoption, and non-transmitted allele designs in presence of simulated components and biases

Using the simulated data, we compare the behaviour of the three designs used in our study to estimate direct and indirect genetic effects. For simplicity's sake our simulations consider one PGS (instead of both *Cog* and *NonCog* PGS). Additionally, we compare to a fourth design which we call "trios" in **Supplementary Figure 9**, in which the phenotype is simply regressed on child and parental PGS. As the simulation results prove, this simple approach gives identical estimates to the non-transmitted allele design, which also uses trios but requires prior identification of segments that are shared and non-shared between the generations.

Supplementary Figure 9 (an extended version of **Figure 3** in the main text) displays the simulation results. The following text discusses the results, focusing on the main estimates of interest – indirect genetic effects of parents.

<u>Prenatal parental indirect genetic effects</u>

We see prenatal effects as a component of interest, rather than as a bias, in estimates of indirect genetic effects. Nonetheless, for consistency with the rest of the simulations which do not consider prenatal indirect genetic effects, the red dashed line in **Supplementary Figure 9** indicates the true postnatal effect only. Simulation results show that the sibling- and trio-based designs capture indirect genetic effects occurring in both prenatal and postnatal periods. In contrast, the adoption design only captures postnatal indirect genetic effects. This is because, for both adoptees and non-adopted individuals, the prenatal environment is provided by the biological mother, so estimated polygenic score-phenotype associations for both adoptees and non-adopted individuals contain prenatal maternal indirect genetic effects. Consequently, computing the indirect genetic effect as the population effect of the polygenic score (β in non-adopted individuals) minus the direct genetic effect (β for adoptees) means that prenatal effects are cancelled out. This result suggests that prenatal indirect genetic effects could partially explain lower estimates of indirect genetic effects from the adoption design compared to the other designs.

<u>Sibling indirect genetic effects</u>

We find that positive sibling effects result in upwardly biased estimates of indirect parental genetic effects. This bias is considerably larger for the sibling design than the adoption and trio designs. Bias in the sibling design is likely to be because positive sibling effects increase the similarity of siblings, reducing the effect of within-sibling polygenic differences [6,7]. Bias in the non-transmitted allele design likely arises because non-transmitted alleles are not only shared with parents, but also partially with (full) siblings, such that βNT might capture sibling as well as parental indirect genetic effects. It is interesting that sibling effects inflate adoption-based estimates despite the fact that adoptees are not genetically related to their siblings. These simulation results lead to the notion that higher estimates of parental indirect genetic effects in the sibling than adoption and non-transmitted allele designs is evidence of sibling indirect

genetic effects. In our empirical data, we do not find differences between sibling- and trio-based estimates of indirect parental genetic effects. Along with our sensitivity analyses, this suggests an absence of sibling genetic effects on educational outcomes in our datasets.

Assortative mating

In our main scenario, which includes substantial error in the GWAS SNP effects used to calculate polygenic scores, so the correlation of GWAS SNP effects and true SNP effects is on average 0.45), we found that the bias from assortative mating in the indirect genetic effect estimate was lower in the adoption design than in the non-transmitted allele and sibling designs. We also tested other scenarios with lower error in the SNP effects used to make the polygenic score. In the scenario with assortative mating but not indirect effects, lower error in the polygenic scores led to decreased bias in estimates from the NT and sibling designs. In the scenario with both assortative mating and indirect effects, with decreasing error in SNP effects, the sibling estimate is consistently biased, but the adoption estimate is more biased and the NT estimate is less biased. Results of other simulations did not change according to the error. We present in the main manuscript the initial results with substantial error as the most conservative example. In real data, we expect this bias due to the combination of error in effect sizes and non-random mating to decrease as GWAS sample sizes increases.

Bias in the sibling design likely arises as the population effect contains assortative mating while the within-sibling effect does not. Bias in the non-transmitted allele design due to assortative mating, which happens due to correlations between parental alleles, is described in Kong et al. 2018 [8]. Interestingly, the bias in the adoption design from assortative mating is zero in the absence of a parental indirect genetic effect, but slightly above zero when a parental indirect genetic effect was also specified. In other words, the presence of parental indirect genetic effects is required for assortative mating to bias estimates from the adoption design. We simulated the same strength of assortative mating for the parents of both adopted and non-adopted individuals, so the result cannot be due to elevated assortment in the latter group (leading to residual assortment in the indirect effect estimate when calculating $\beta_{non\text{-}adopted}$ - $\beta_{adopted}$). Such differences could exist in the real data, but there is scarce and inconsistent evidence regarding assortment in biological parents of adoptees versus other parents [9,10]. Overall, the results suggest that assortative mating could explain lower estimates of indirect genetic effects from the adoption design compared to the other designs, but may depend on the level of noise in the GWAS effects.

Population stratification

Simulation results show that estimates of parental indirect genetic effects based on the adoption design capture less bias from population stratification than sibling- and trio-based designs. In the sibling design, the parental indirect genetic effect is estimated as the population effect minus the direct within-family effect of the polygenic score. This means that the indirect genetic effect is likely to be inflated by population stratification, as this is captured in the population effect but not the within-family effect. Also, the effects of the non-transmitted allele PGS are influenced by population stratification, so the indirect genetic effect estimate is inflated. In contrast, population stratification only biases indirect genetic effect estimates from

the adoption design to a small extent. Assuming that population stratification is similar in adoptees and non-adopted individuals, its effect will cancel out when estimating the indirect genetic effect as β*non-adopted* - β*adopted*. The assumption of equal population stratification and assortative mating bias in adopted and non-adopted groups cannot be tested due to the lack of parental data in UKB, but is bolstered by the simulation results, and by the fact that both adoptees and non-adopted individuals are from British ancestry. Our simulation results suggest that population stratification partly explains the lower estimates of indirect genetic effects from the adoption design compared to the other designs in our empirical study.

Supplementary Figures

Supplementary Figure 1. GWAS-by-subtraction Genomic-SEM model, reproduced from Demange et al. 2021

Procedure identical to Demange et al [11], description repeated verbatim for convenience: Cholesky model as fitted in Genomic SEM, with path estimates for a single SNP included as illustration. SNP, Cognitive performance (CP) and Educational attainment (EA) are observed variables based on GWAS summary statistics. The genetic covariance between CP and EA is estimated based on GWAS summary statistics for CP and EA. The model is fitted to a 3x3 observed variance-covariance matrix (i.e. SNP, CP, EA). Cog and Non-Cog are latent (unobserved) variables. The covariances between CP and EA and between Cog and NonCog are fixed to 0. The variance of the SNP is fixed to the value of 2pq (p = reference allele frequency, q = alternative allele frequency, based on 1000 Genomes phase 3). The residual variances of CP and EA are fixed to 0, so that all variance is explained by the latent factors. The variances of the latent factors are fixed to 1. The latent variables were then regressed on each SNP that met QC criteria. Reproduced from Demange, P.A., Malanchini, M., Mallard, T.T. et al. Investigating the genetic architecture of noncognitive skills using GWAS-by-subtraction. *Nat Genet* 53, 35–44 (2021). Springer Nature. https://doi.org/10.1038/s41588-020-00754-2

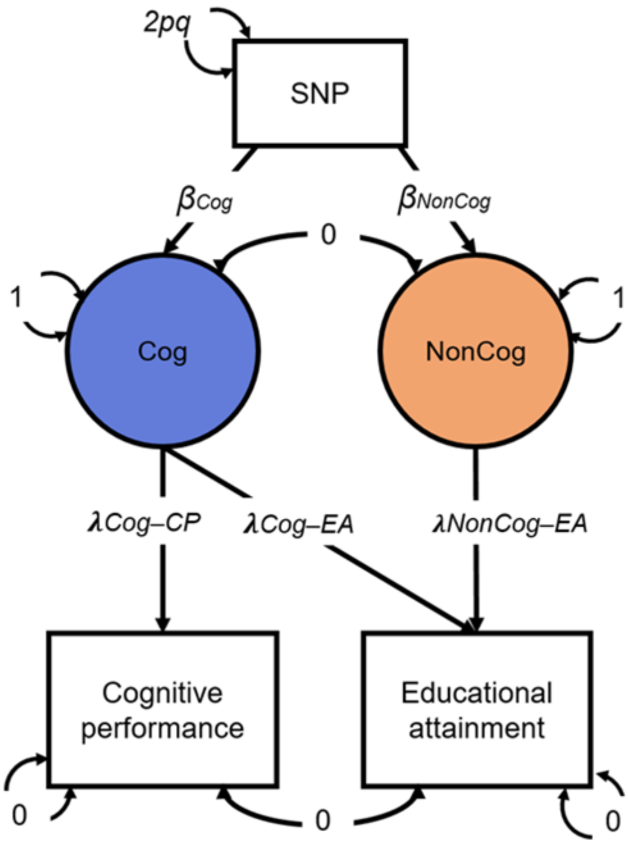

Supplementary Figure 2. Birthplace coordinates for adoptees and non-adopted individuals

Note: adopted (Left) and non-adopted (Right); colours = regions obtained by k-means clustering of coordinates.

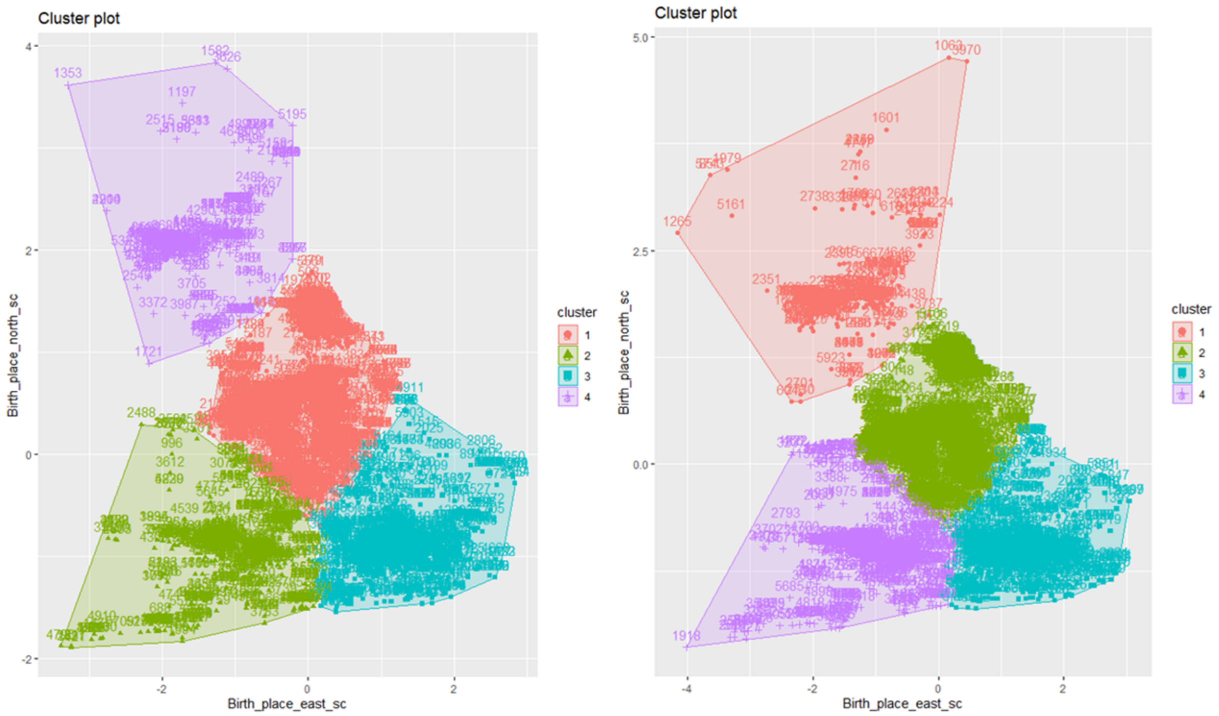

Supplementary Figure 3. All direct and indirect genetic effect estimates

Estimates of direct and indirect effects of *NonCog* and *Cog* PGS for every condition. Method is represented by a symbol, sample and outcome are represented by a color, bars are 95% CIs. Trio = Non-transmitted design. Precise values are presented in **Supplementary Data 3.** Moving from left to right on the x-axis sample sizes were: 1631 (Sibling NTR CITO), 1526 (Non-transmitted NTR CITO), 3163 (Sibling NTR EA), 2534 (Non-transmitted NTR EA), 2862 (Sibling TEDS 12yo), 4796 (Sibling TEDS GCSE), 6407 adopted and 6500 non-adopted (Adoption UKB EA), and 39500 (Sibling UKB EA).

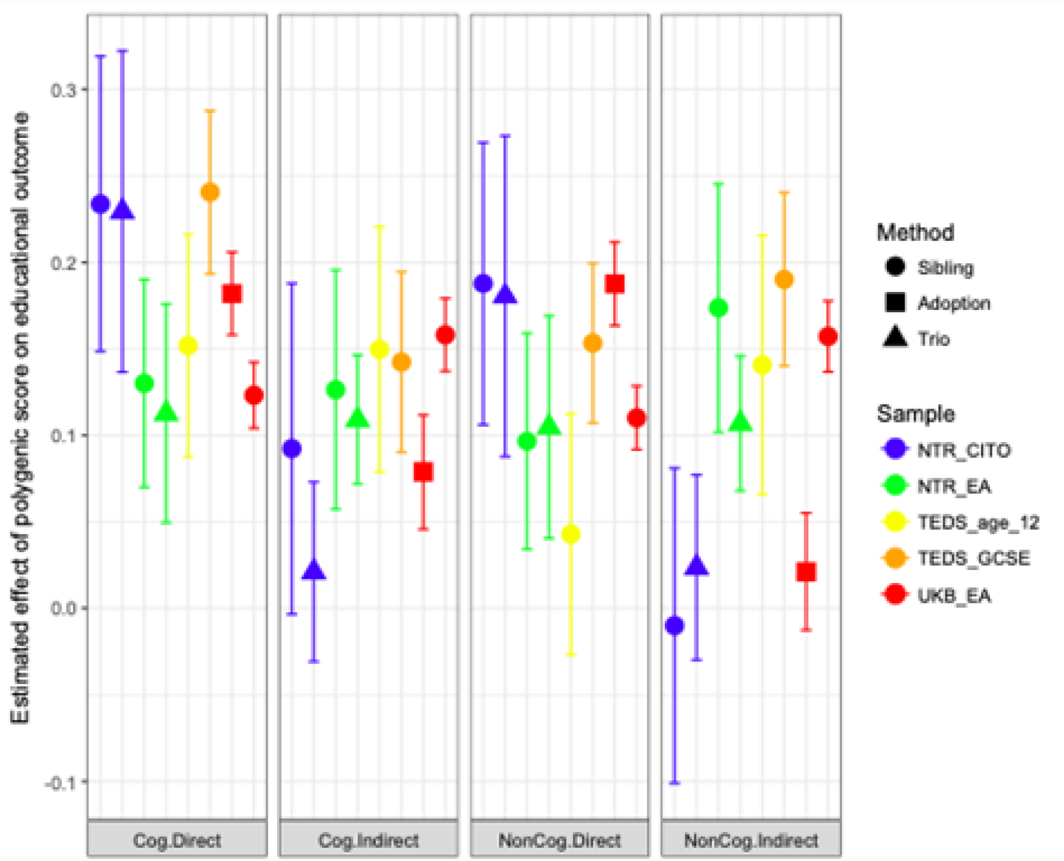

Supplementary Figure 4. Population genetic effects

Estimates of population effects of *NonCog* (orange) and *Cog* (blue) PGS for every condition grouped by method. X axis ticks indicate the sample (NTR, TEDS and UKB) and outcomes (CITO is age 12 achievement in NTR, 12yo is age 12 teacher rated achievement in TEDS, GCSE is age 16 achievement in TEDS and EA), bars are 95% CIs. Moving from left to right on the x-axis sample sizes were: 1631 (Siblings CITO), 3163 (Siblings EA), 2862 (Siblings 12yo), 4796 (Siblings GCSE), 39500 (Siblings UKB EA), 6407 adopted and 6500 non-adopted (Adoption EA), 1526 (Non-transmitted CITO) and 2534 (Non-transmitted EA). Full estimates are shown in **Supplementary Data 3.**

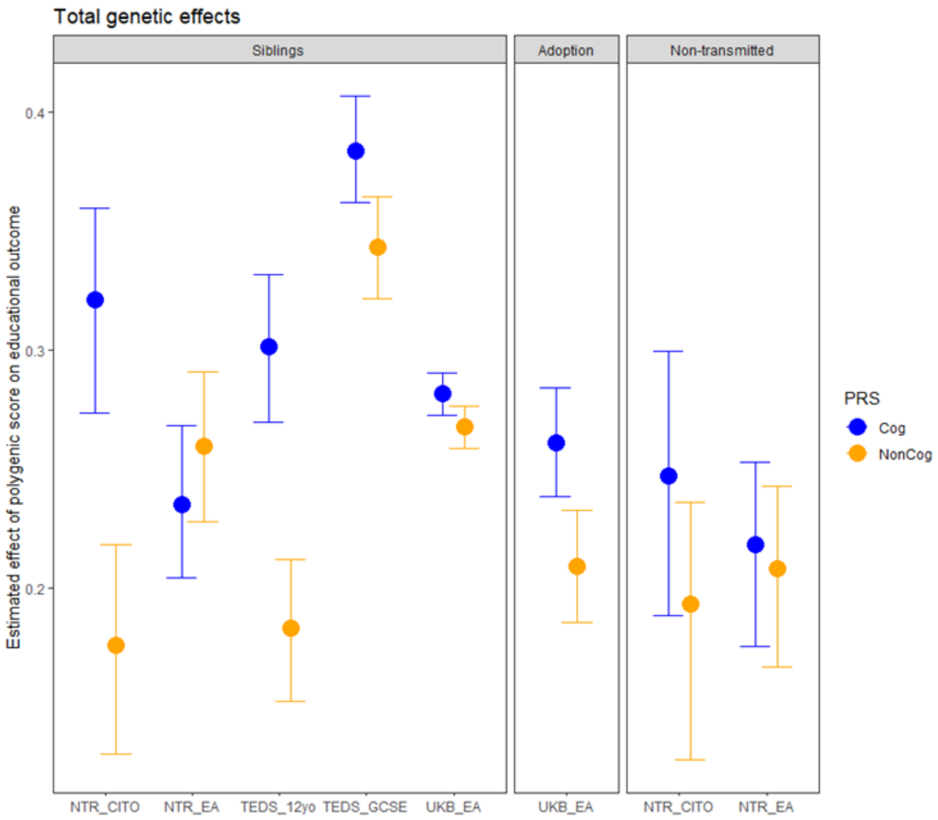

Supplementary Figure 5. Ratios indirect effect/population effect

Estimates of the ratio of the indirect effects on the population effects of *NonCog* (orange) and *Cog* (blue) PGS for every condition grouped by method. X axis ticks indicate the sample (NTR, TEDS and UKB) and outcomes (CITO is age 12 achievement in NTR, 12yo is age 12 teacher rated achievement in TEDS, GCSE is age 16 achievement in TEDS and EA); bars are 95% CIs. Moving from left to right on the x-axis sample sizes to estimate the indirect and total effects on which these ratios are based were: 1631 (Siblings CITO), 3163 (Siblings EA), 2862 (Siblings 12yo), 4796 (Siblings GCSE), 39500 (Siblings UKB EA), 6407 adopted and 6500 non-adopted (Adoption EA), 1526 (Non-transmitted CITO) and 2534 (Non-transmitted EA). Full estimates are in **Supplementary Data 3.**

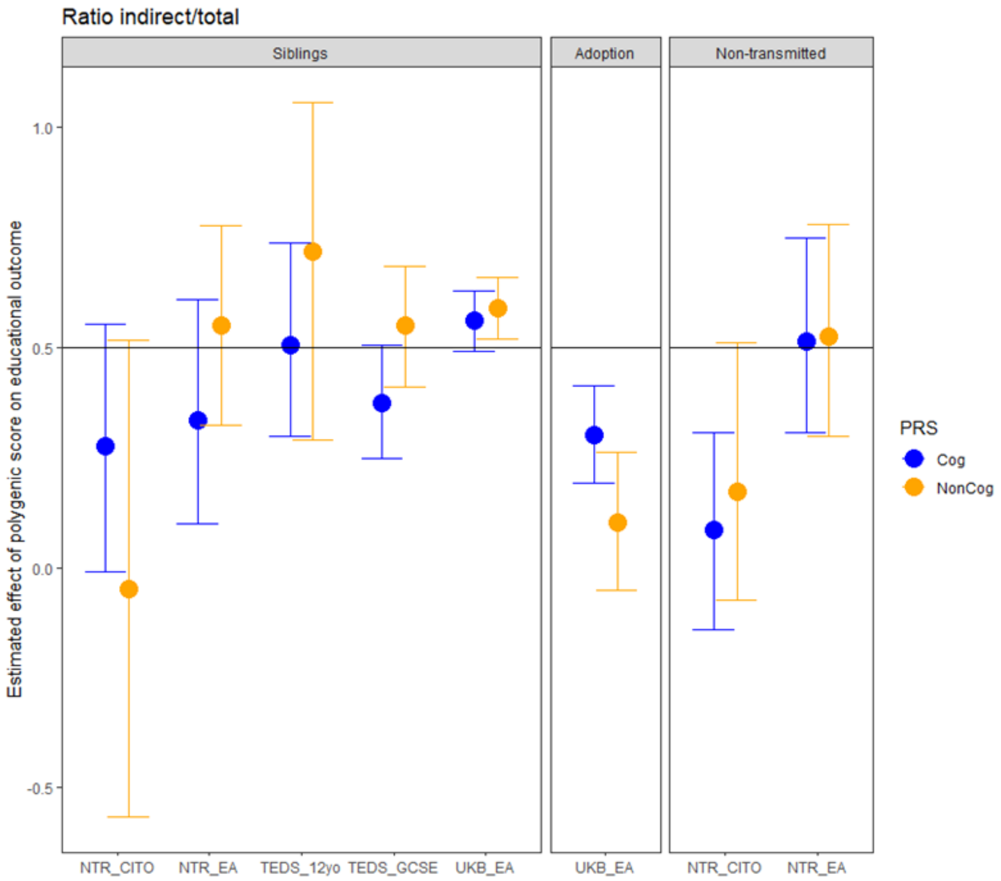

Supplementary Figure 6. Effect of the *NonCog* and *Cog* polygenic score on educational outcomes in monozygotic and dizygotic twins in TEDS and NTR

A. Effect of *NonCog* PGS on educational outcomes in MZ vs DZ.

B. Effect of *Cog* PGS on educational outcomes in MZ vs DZ.

Y axis represents the beta coefficient of the regression of the *NonCog/Cog* PGS on educational outcomes. In blue are results in dizygotic twins and green in monozygotic twins. Bars represent the 95% CIs of the estimate. X axis ticks indicate the individual zygosity (MZ: monozygotic twin; DZ: dizygotic twin) and outcomes (age12 is age 12 teacher rated achievement in TEDS, age16 is age 16 achievement in TEDS, EA is educational attainment in NTR and CITO is age 12 achievement in NTR). Moving from left to right on the x-axis sample sizes were 2709, 546, 2709, 546, 865, 818, 1369, 1600. Values are in Supplementary Data 8.

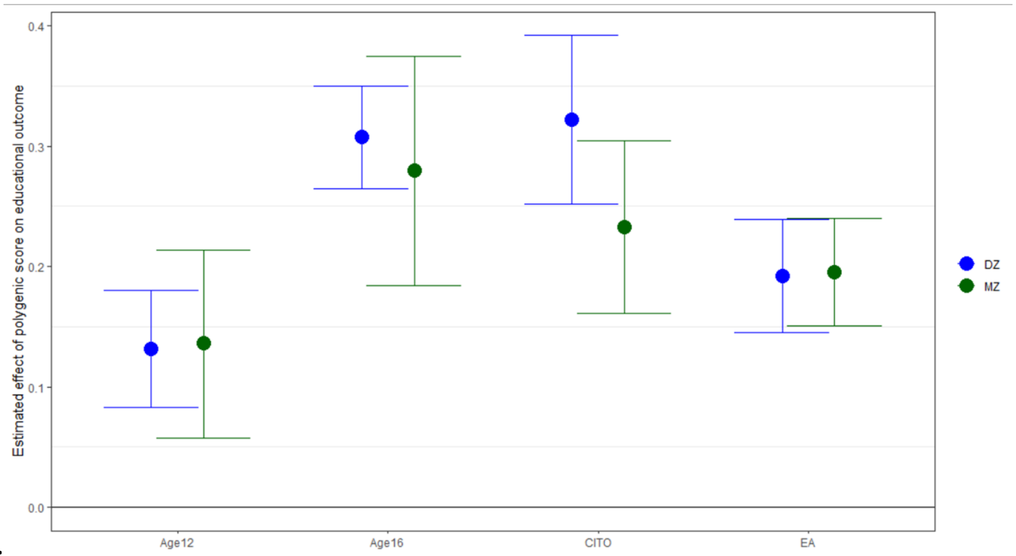

A.

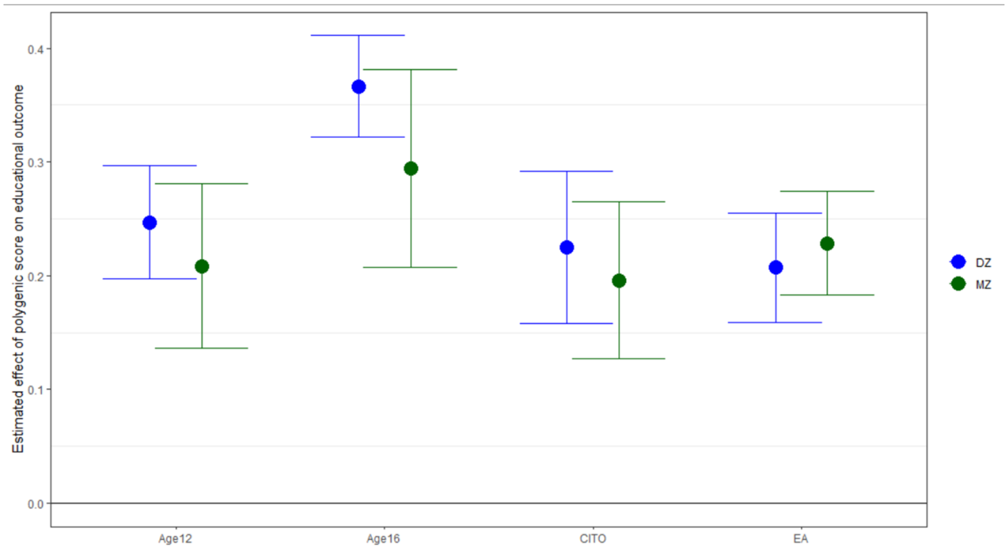

B.

Supplementary Figure 7. Estimated effect of NonCog and Cog PGS on Educational Attainment in UK Biobank depending on the number of siblings (adopted or full-siblings) of the individual

We look at the effect of *NonCog* and *Cog* PGS in non-adopted and adopted individuals (the latter group providing a control scenario since they are not genetically related to their sibling(s)). Blue dots represent the estimates of the *Cog* polygenic score effect (Beta) on educational attainment, bars are 95% CI. In orange are *NonCog* estimates. We draw the linear regression model between these estimates (blue and orange lines). Moving from left to right on the x-axis sample sizes were 2357, 1725, 709, 336, 175 for adoptees, and 830, 2111, 1599, 926, 436 for non-adopted individuals. Values are in Supplementary Data 9.

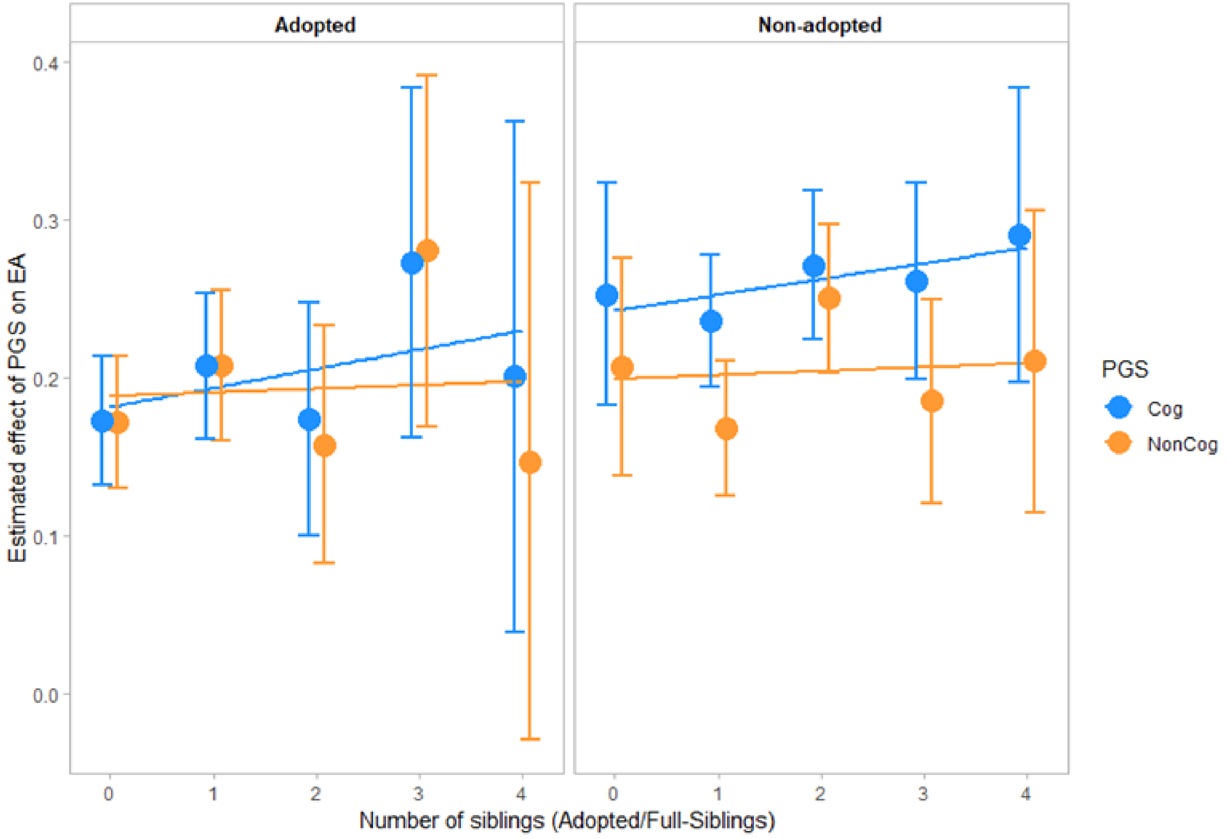

Supplementary Figure 8. Estimation of the direct and parental indirect genetic effects in simulated data with different implementations of the sibling design

Estimates of parental direct and indirect genetic effects from two implementations of the sibling design, based on data simulated to include indirect genetic effects or not. Boxplots of 100 replicates based on a simulated sample of 20,000 families. The center line represents the median, the box limits are the 1st and 3rd quartile, and the whiskers reach the maximum value within 1.5 times the interquartile range. Outlying values are not represented. For clarity, the red line benchmarks the true simulated *postnatal* parental indirect effect, although we note that *prenatal* parental genetic effects are a component rather than a bias of the parental indirect genetic effect.

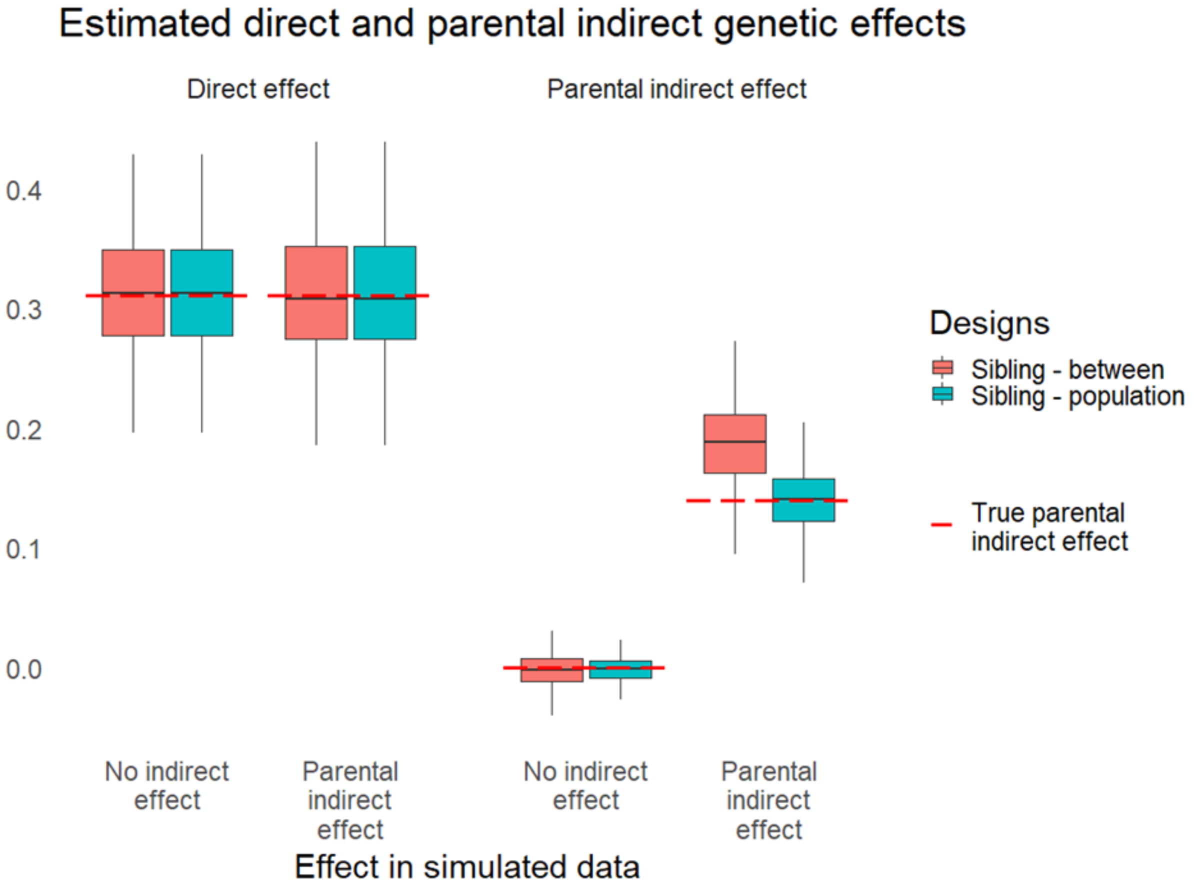

Supplementary Figure 9. Simulation results: Comparison of sibling, adoption, and non-transmitted allele designs in presence of components and biases

Estimates of parental direct and indirect genetic effects from the three designs, based on data simulated to include different components and biases. Boxplots of 100 replicates based on a simulated sample of 20,000 families. The center line represents the median, the box limits are the 1st and 3rd quartile, and the whiskers reach the maximum value within 1.5 times the interquartile range. Outlying values are not represented. For clarity, the red line benchmarks the true simulated *postnatal* parental indirect effect, although we note that *prenatal* parental genetic effects are a component rather than a bias of the parental indirect genetic effect.

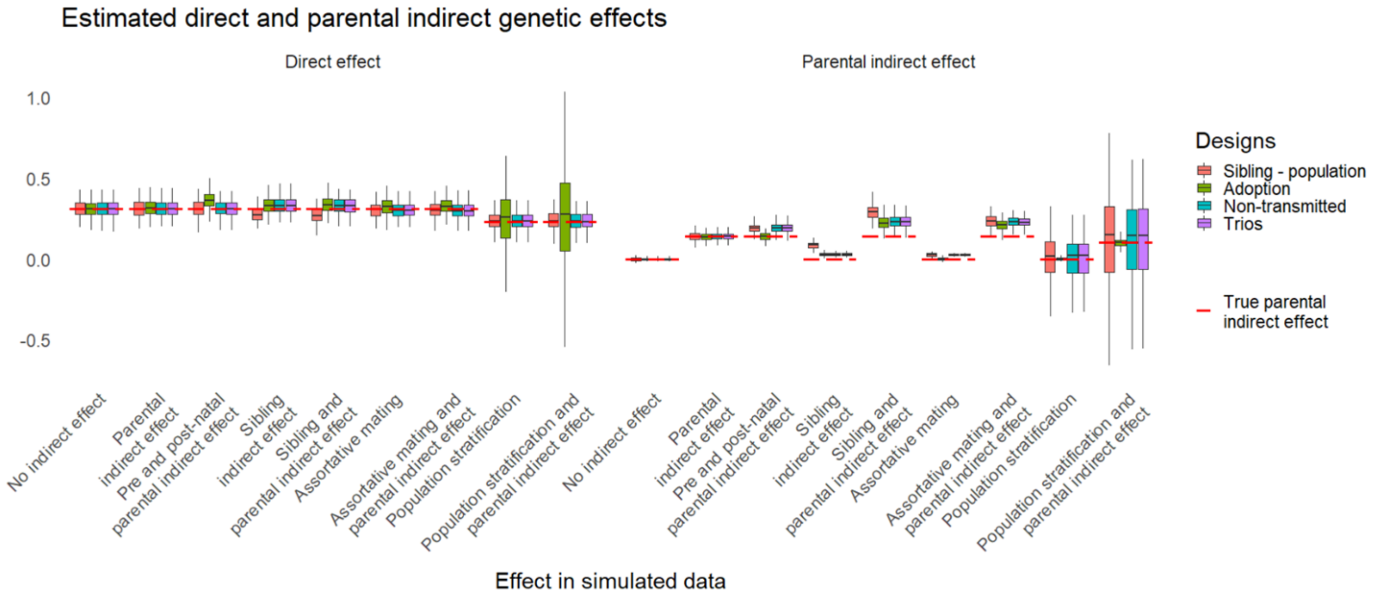

Supplementary References

1. Selzam, S. *et al.* Comparing Within- and Between-Family Polygenic Score Prediction. *Am. J. Hum. Genet.* **105,** 351–363 (2019).
2. Demange, P. & Cheesman, R. Estimating effects of parents' cognitive and non-cognitive skills on offspring education using polygenic scores, GitHub. *Zenodo* (2022). doi:10.5281/zenodo.6581326
3. Carlin, J. B., Gurrin, L. C., Sterne, J. A., Morley, R. & Dwyer, T. Regression models for twin studies: a critical review. *Int. J. Epidemiol.* **34,** 1089–1099 (2005).
4. Howe, L. J. *et al.* Within-sibship GWAS improve estimates of direct genetic effects. *BioRxiv* (2021). doi:10.1101/2021.03.05.433935
5. Cheesman, R. *et al.* Comparison of Adopted and Nonadopted Individuals Reveals Gene-Environment Interplay for Education in the UK Biobank. *Psychol. Sci.* **31,** 582–591 (2020).
6. Boardman, J. D. & Fletcher, J. M. To cause or not to cause? That is the question, but identical twins might not have all of the answers. *Soc. Sci. Med.* **127,** 198–200 (2015).
7. Kohler, H.-P., Behrman, J. R. & Schnittker, J. Social science methods for twins data: integrating causality, endowments, and heritability. *Biodemography Soc Biol* **57,** 88–141 (2011).
8. Kong, A. *et al.* The nature of nurture: Effects of parental genotypes. *Science* **359,** 424–428 (2018).
9. Plomin, R., DeFries, J. C. & Roberts, M. K. Assortative mating by unwed biological parents of adopted children. *Science* **196,** 449–450 (1977).
10. Ho, H., Plomin, R. & DeFries, J. C. Selective placement in adoption. *Soc Biol* **26,** 1–6 (1979).
11. Demange, P. A. *et al.* Investigating the genetic architecture of noncognitive skills using GWAS-by-subtraction. *Nat. Genet.* **53,** 35–44 (2021).
