# Supplementary material for: Estimating effects of parents’ cognitive and non-cognitive skills on offspring education using polygenic scores
Source: Nat Commun. 2022 Aug 23;13:4801. doi: 10.1038/s41467-022-32003-x (PMC9399113; doi:10.1038/s41467-022-32003-x)
Supplement: Supplementary file 3 — Description of Additional Supplementary Files [file 41467_2022_32003_MOESM3_ESM.pdf]

Description of Additional Supplementary Files

File Name: Supplementary Data 1
Description: Information on the GWAS.

File Name: Supplementary Data 2
Description: Samples descriptive statistics.

File Name: Supplementary Data 3
Description: Bootstrapped results of the direct, indirect, population effects and ratio indirect/population for each condition.

File Name: Supplementary Data 4
Description: Meta-analyses results.

File Name: Supplementary Data 5
Description: Z-tests to compare direct and indirect estimates and Cog and NonCog indirect/direct ratios.

File Name: Supplementary Data 6
Description: Estimations of correlations to assess assortative mating.

File Name: Supplementary Data 7
Description: Estimation of indirect sibling effect with Sibling PGS in NTR trio data.

File Name: Supplementary Data 8
Description: PGS predictions of educational outcomes in MZ-only and DZ-only samples.

File Name: Supplementary Data 9
Description: PGS predictions of EA in Adoptees and Non-adopted (control) per number of siblings.

File Name: Supplementary Data 10
Description: Pearson's correlations of PGS.

File Name: Supplementary Data 11
Description: Comparison of demographic and early-life characteristics of the adopted and non-adopted samples.
