# Supplementary material for: Estimating effects of parents’ cognitive and non-cognitive skills on offspring education using polygenic scores
Source: Nat Commun. 2022 Aug 23;13:4801. doi: 10.1038/s41467-022-32003-x (PMC9399113; doi:10.1038/s41467-022-32003-x)
Supplement: Supplementary file 5 — Reporting Summary [file 41467_2022_32003_MOESM5_ESM.pdf]

Corresponding author(s): Perline Demange, Rosa Cheesman

Last updated by author(s): 27/05/2022

Reporting Summary

Nature Portfolio wishes to improve the reproducibility of the work that we publish. This form provides structure for consistency and transparency in reporting. For further information on Nature Portfolio policies, see our Editorial Policies and the Editorial Policy Checklist.

Statistics

For all statistical analyses, confirm that the following items are present in the figure legend, table legend, main text, or Methods section.

n/a	Confirmed	
☐	☒	The exact sample size (n) for each experimental group/condition, given as a discrete number and unit of measurement
☐	☒	A statement on whether measurements were taken from distinct samples or whether the same sample was measured repeatedly
☐	☒	The statistical test(s) used AND whether they are one- or two-sided<br>*Only common tests should be described solely by name; describe more complex techniques in the Methods section.*
☐	☒	A description of all covariates tested
☐	☒	A description of any assumptions or corrections, such as tests of normality and adjustment for multiple comparisons
☐	☒	A full description of the statistical parameters including central tendency (e.g. means) or other basic estimates (e.g. regression coefficient) AND variation (e.g. standard deviation) or associated estimates of uncertainty (e.g. confidence intervals)
☐	☒	For null hypothesis testing, the test statistic (e.g. F, t, r) with confidence intervals, effect sizes, degrees of freedom and P value noted<br>*Give P values as exact values whenever suitable.*
☒	☐	For Bayesian analysis, information on the choice of priors and Markov chain Monte Carlo settings
☒	☐	For hierarchical and complex designs, identification of the appropriate level for tests and full reporting of outcomes
☐	☒	Estimates of effect sizes (e.g. Cohen's d, Pearson's r), indicating how they were calculated

Our web collection on statistics for biologists contains articles on many of the points above.

Software and code

Policy information about availability of computer code

Data collection	No software was used for data collection.
Data analysis	Meta-analysis of summary statistics was performed with Metal, release 2011-03-25.<br>The GWAS-by-subtraction was performed with GenomicSEM v0.0.2, in R 3.4.3.<br>Genotyping platforms are described in Methods. PCA was conducted with flashPCA v2 and Smartpca v6.40. Identification of the transmitted and non-transmitted genotypes was done with Plink 1.07 and ShapeIT software v2.r904. NTR data was phased using Eagle v2.4.1 and then imputed to 1000 Genomes and Topmed using Minimac3-omp v2.10, VCF files were merged with Bcftools 1.9, and DNA identity-by-descent state was estimated using the Plink 1.9 and King 2.1.6 programs.<br>Polygenic scores weights were calculated using LDpred v1.0.0, scores were built with Plink 1.9 and 2. Polygenic scores predictions and other analyses were run in R 3.6. Sibship identification in UKBiobank was performed using bash. Ratio and LDscore intercept was assessed with the ldsc software.<br>All scripts used to run the analyses (empirical and simulated) are available at: https://github.com/PerlineDemange/GeneticNurtureNonCog

For manuscripts utilizing custom algorithms or software that are central to the research but not yet described in published literature, software must be made available to editors and reviewers. We strongly encourage code deposition in a community repository (e.g. GitHub). See the Nature Portfolio guidelines for submitting code & software for further information.

Data

Policy information about availability of data

All manuscripts must include a data availability statement. This statement should provide the following information, where applicable:

- Accession codes, unique identifiers, or web links for publicly available datasets
- A description of any restrictions on data availability
- For clinical datasets or third party data, please ensure that the statement adheres to our policy

Summary Statistics of Cog and NonCog used in this paper are available upon request. Summary Statistics of cognitive performance from the COGENT cohort, of EA excluding NTR and UKBiobank cohorts are available upon request to the communicating author of these papers.
Researchers can apply for access to TEDS data: https://www.teds.ac.uk/researchers/teds-data-access-policy
For UK Biobank dataset access, see: https://www.ukbiobank.ac.uk/using-the-resource/.
Netherlands Twin Register data may be accessed, upon approval of the data access committee, email: ntr.datamanagement.fgb@vu.nl

Field-specific reporting

Please select the one below that is the best fit for your research. If you are not sure, read the appropriate sections before making your selection.

☐ Life sciences ☒ Behavioural & social sciences ☐ Ecological, evolutionary & environmental sciences

For a reference copy of the document with all sections, see nature.com/documents/nr-reporting-summary-flat.pdf

Behavioural & social sciences study design

All studies must disclose on these points even when the disclosure is negative.

Study description	Quantitative research
Research sample	We used one biobank (UK Biobank) and two longitudinal twin studies (NTR, TEDS) datasets. These are genotyped cohorts including family members and detailed educational information. These cohorts were necessary for our aim to estimate indirect genetic effects and comparing estimates between methods. Demographic statistics of our samples are detailed in Supplementary Table 2. TEDS is fairly UK-representative (biased towards higher educated families). UK Biobank is not representative of the general UK population on a variety of characteristics. The genotyped sample of NTR is also biased towards more educated participants than the general dutch population. Further description of the UKBiobank, TEDS and NTR can be found in the method section.
Sampling strategy	Our sample strategy was to collect the maximum sample of participants from european ancestry with genotype and educational phenotype information, and relatedness structure (siblings or parents-child). Our sample size ranged from 1631 to 39500 individuals, which is above the sample required for polygenic score analyses (~minimum of several hundred).
Data collection	We used previously collected data, with collection and recruitment described elsewhere (Rimfeld et al. 2019, Ligthart et al. 2019, https://www.ukbiobank.ac.uk/key-documents/, see Methods). Phenotypes used were recorded via questionnaires. Data collection was prior to initiation of this study. The current analysts were not blinded to study hypotheses.
Timing	UK Biobank data is collected since 2007, TEDS data is collected since 1994 and NTR data since 1986. Data collection is still ongoing. Analyses started in 2020 and we therefore included in the analysed data participants who reported their education outcomes before 2020.
Data exclusions	We excluded participants on the grounds of missingness for phenotypic and genomic variables of study. We excluded participants with non-european ancestry due to the use of polygenic scores based on european ancestry.
Non-participation	Not applicable, we do not have data on people who were invited to participate in UKBiobank, TEDS and NTR, but did not
Randomization	Not applicable, participants were not allocated into experimental groups.

Reporting for specific materials, systems and methods

We require information from authors about some types of materials, experimental systems and methods used in many studies. Here, indicate whether each material, system or method listed is relevant to your study. If you are not sure if a list item applies to your research, read the appropriate section before selecting a response.

Materials & experimental systems

n/a	Involved in the study
☒	☐ Antibodies
☒	☐ Eukaryotic cell lines
☒	☐ Palaeontology and archaeology
☒	☐ Animals and other organisms
☐	☒ Human research participants
☒	☐ Clinical data
☒	☐ Dual use research of concern

Methods

n/a	Involved in the study
☒	☐ ChIP-seq
☒	☐ Flow cytometry
☒	☐ MRI-based neuroimaging

Human research participants

Policy information about studies involving human research participants

Population characteristics

Population characteristics are described in Supplementary Table 2.

Recruitment

NTR recruits approximately 40% of new-born twins or higher-order multiples in the Netherlands for longitudinal research. Adult twins are registered with the NTR through several approaches (i.e. recruitment through city council offices in the Netherlands, advertising in NTR newsletters and the internet). Parents, siblings, spouses and offspring of adult twins are also invited to take part. Participants receive a survey every 2 to 3 years with questions on, amongst others, health, personality, and lifestyle. The NTR has also been collecting genotype data in both children and adults in several large projects. More details concerning the NTR's data collection are described elsewhere (Ligthart et al. 2019).
TEDS: All twins born between 1994 and 1996 in England and Wales, as identified through birth records, were invited via their parents to participate. More details are available in Rimfeld et al. 2019
UKBiobank recruited 500,000 participants aged 40-69 years between 2006-2010. The full recruitment process is described here: https://www.ukbiobank.ac.uk/wp-content/uploads/2011/11/UK-Biobank-Protocol.pdf

Ethics oversight

Ethical approval for NTR was provided by the Central Ethics Committee on Research Involving Human Subjects of the VU University Medical Center, Amsterdam, and Institutional Review Board certified by the U.S. Office of Human Research Protections (IRB number IRB-2991 under Federal-wide Assurance-3703; IRB/institute codes 94/105, 96/205, 99/068, 2003/182, 2010/359).
UKBiobank has received ethical approval from the National Health Service North West Centre for Research Ethics Committee (reference: 11/NW/0382).
TEDS's Project approval was granted by King's College London's ethics committee for the Institute of Psychiatry, Psychology and Neuroscience PNM/09/10–104.

Note that full information on the approval of the study protocol must also be provided in the manuscript.
